# A curated database of cyanobacterial strains relevant for modern taxonomy and phylogenetic studies

**DOI:** 10.1038/sdata.2017.54

**Published:** 2017-04-25

**Authors:** Vitor Ramos, João Morais, Vitor M. Vasconcelos

**Affiliations:** 1 CIIMAR/CIMAR—Interdisciplinary Centre of Marine and Environmental Research, University of Porto, Terminal de Cruzeiros do Porto de Leixões, Av. General Norton de Matos s/n, Matosinhos 4450-208, Portugal; 2 Faculdade de Ciências, Universidade do Porto, Rua do Campo Alegre, Porto 4169-007, Portugal

**Keywords:** Phylogeny, Taxonomy, Data integration, Classification and taxonomy, Bacteria

## Abstract

The dataset herein described lays the groundwork for an online database of relevant cyanobacterial strains, named CyanoType (http://lege.ciimar.up.pt/cyanotype). It is a database that includes categorized cyanobacterial strains useful for taxonomic, phylogenetic or genomic purposes, with associated information obtained by means of a literature-based curation. The dataset lists 371 strains and represents the first version of the database (CyanoType v.1). Information for each strain includes strain synonymy and/or co-identity, strain categorization, habitat, accession numbers for molecular data, taxonomy and nomenclature notes according to three different classification schemes, hierarchical automatic classification, phylogenetic placement according to a selection of relevant studies (including this), and important bibliographic references. The database will be updated periodically, namely by adding new strains meeting the criteria for inclusion and by revising and adding up-to-date metadata for strains already listed. A global 16S rDNA-based phylogeny is provided in order to assist users when choosing the appropriate strains for their studies.

## Background & Summary

Strains held in culture collections are pivotal for comparative purposes in current taxonomic or phylogenetic studies of prokaryotes in general, and cyanobacteria in particular. In recent years, the number of new cyanobacterial genera established by a modern polyphasic taxonomy (i.e., following a combination of different techniques) has greatly increased^1^, resulting in the designation of several new Type strains (i.e., an isolate based on which the author describes a new species or genus; it is often the holotype specimen itself).

Concerning nomenclature, cyanobacteria (formerly known as blue-green algae) are a special case among the prokaryotes, since they are ruled either by the International Code of Nomenclature for algae, fungi, and plants (ICN; formerly the International Code of Botanical Nomenclature, ICBN) or by the International Code of Nomenclature of Prokaryotes (ICNP, formerly the International Code of Nomenclature of Bacteria, ICNB). Nomenclature rules governed by these two entities are converging^1–5^ but due to this duality two general types of systematics still exists: the more ancient botanical/phycological classification scheme and the bacteriological scheme^4^. Available keys for the identification of cyanobacteria are mostly based on the botanical system proposed by Geitler in 1932 (ref. 6), including the key present in the pioneering bacteriological system of Stanier and colleagues^2,7^. One important classification system followed by microbiologists is the Bergey’s Manual of Systematic Bacteriology, often confused as an ‘official classification’, which is not the case^8^. The manual classifies the cyanobacteria in ‘form genera’ which, in turn, are divided into clusters or subclusters^9^. For each (sub)cluster at least one Reference strain is assigned. This strain category, as presented in the manual, should not be confused with being a Type strain (though some of them effectively are). Moreover, the term ‘form genus’ has no standing under the Bacteriological or under the Botanical Codes of Nomenclature^2^ and the authors of the cyanobacterial section of the manual early admitted that the proposed classification is a temporary one^2,9^. Despite these taxonomic issues, the Bergey’s Manual is an important body of work, since it systematizes, lists and characterizes a good number of cyanobacterial strains, most of which are widely used as reference in phylogenetic studies.

Several new taxa that have been recently established emerge from taxonomic revisions of ‘classical’ botanical genera, which have been described primarily by their morphological features. Most of them are in fact polyphyletic, as depicted from 16S rRNA gene-based phylogenies using strains assigned to different species of such genera^1,4,9^. Since the pioneering work of Carl Woese, George Fox and colleagues^10–12^, the 16S rRNA gene became, and still is, the most important and widely used molecular marker for the identification of prokaryotes. However, its resolving power at species level is low^13^, and should therefore be employed to obtain identifications at the genus level. Nonetheless, its appropriateness for phylogenetic-based classifications was again demonstrated more recently. Shih *et al.*^14^ have demonstrated, following a phylogenomic approach involving 54 cyanobacterial genomes, that the 16S rRNA phylogeny is highly congruent with that obtained from a concatenation of 31 conserved proteins. Thus, it is likely that the 16S rRNA gene will continue to be the standard molecular marker for proposing new cyanobacterial genera^1^. The emergence of genome-based taxonomy^15^ approaches, however, renders genome-sequenced strains increasingly important to the field.

Due to the above-mentioned issues, choosing the proper strains to include in taxonomic, phylogenetic or comparative genomic studies on cyanobacteria is very often a challenging task. In order to overcome this difficulty, we introduce the curated dataset of CyanoType v.1, a database with an extensive list of relevant cyanobacterial strains classified by importance category, i.e., with the indication about being a Type strain, a Reference strain *sensu* Bergey’s Manual, and/or a strain having its genome sequenced. The dataset encompasses different types of metadata (e.g., strain synonymy and/or co-identity), including a reference list for each strain. In order to help users in their process of selecting strains, we provide two 16S rDNA-based phylogenetic trees for guidance. The main phylogenetic tree and the information for each strain included in the dataset is available in the searchable, online database at http://lege.ciimar.up.pt/cyanotype.

## Methods

**Figure 1:**
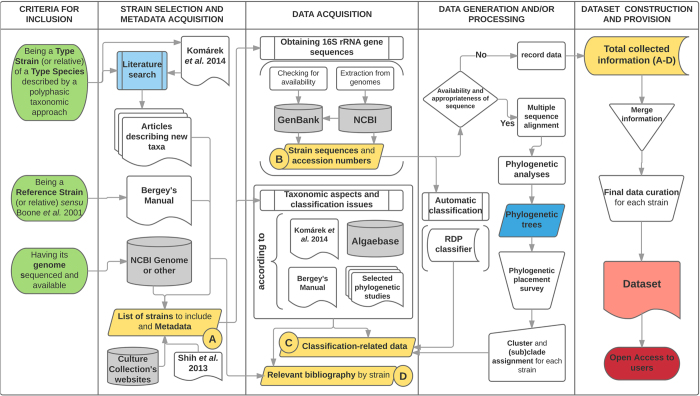
Diagram illustrating the workflow followed during the construction and release of the dataset (standard flowchart symbols were used).

Figure 1 illustrates the workflow followed for the literature and database searches performed in this study.

### Data acquisition

We initially established the criteria for inclusion of cyanobacterial strains in the dataset. We have considered three main groups of strains to be included, representing different levels of importance from the taxonomic point of view: (1) strains that were used as Type strains for the proposal or establishment of a new taxon by mean of a modern, polyphasic taxonomic approach^1^, (2) strains that are included as Reference strains in Bergey's Manual of Systematic Bacteriology^9^, and (3) strains that have their genome sequenced and publicly available. Following these criteria, and through literature and online database searches, we have obtained a list of relevant strains categorized by taxonomic importance.

Literature searches for Type strains were firstly guided by information included in the work of Komárek *et al.*^1^, which lists the cyanobacterial genera for which the holotype (i.e., Type species) was described using a modern, polyphasic taxonomic approach. To avoid missing any Type strain (e.g., strains from new genera arising from later studies than those included in Komárek *et al.*^1^) we have performed complementary searches in literature databases such as ISI Web of Science, Scopus, PubMed and Google Scholar using the following Boolean search string: ((cyanobact* OR cyanophy*) AND ((gen. nov. OR gen. et sp. nov.) OR ‘new genus’ OR ‘novel genus’ OR ‘new genera’ OR ‘novel genera’) for the fields [Title, Abstract, Keywords]. Duplicate articles were eliminated. We then fully examined the search results to evaluate the suitability of the articles for our research.

The dataset also includes Reference strains from all clusters or subclusters defined in the Bergey’s Manual^9^, and known relatives (as indicated in the Manual) if the 16S rRNA gene sequences for the Reference strains were not available. The number of strains fitting in each category are summarized in Table 1. A full description of each category type can be found in the Data Records section (see Strain_Category). We have also—as was done for strains—performed literature and online database searches for data and metadata acquisition. For instance, we manually performed data mining in public molecular (e.g., NCBI) and taxonomic (e.g., AlgaeBase, http://www.algaebase.org/) databases and searches on websites of Culture Collections.

Finally, strains with available cyanobacterial genomes were also included in the dataset. The work of Shih *et al.*^14^ was used as a reference first list. To obtain the full list, we have then used the Assembly database and other NCBI resources (e.g., Genome, Genome BLAST, BLASTn) in order to search for genomes and to obtain accession numbers and 16S rRNA gene nucleotide sequences from the strains. The search term Cyanobacteria (Taxonomy ID: 1,117) was used to obtain the list of available cyanobacterial genomes. For our study, we have considered 251 out of 372 strains having their genomes available in NCBI (until the end of 2015). Missing strains are *Prochlorococcus* spp. which were not included in the dataset due to overrepresentation and phylogenetic redundancy. Even so, the dataset comprises 28 representatives, by far the most represented genus (Data Citation 1).

All strains with available 16S rRNA gene nucleotide sequences were then subjected to a phylogenetic study (see Subsection ‘Phylogenetic analyses’ below). First, in order to obtain the sequences, we have performed Boolean searches in the NCBI Nucleotide database (which includes GenBank). For some strains it was necessary to extract the sequences by mining their genome. Accession numbers were recorded in the dataset (Data Citation 1). Adequacy of the sequences length for multiple alignment and further analyses was then checked (see Subsection ‘Phylogenetic analyses’). Additionally, the 16S rRNA gene sequences were submitted to the automatic RDP Naive Bayesian rRNA Classifier v2.6 (ref. 16) pipeline. Strains were ranked and the hierarchical classification result recorded (Data Citation 1).

Moreover, we have classified the strains at higher taxonomic levels (Order and Family) and verified the nomenclatural status of taxon names according to taxonomic concepts followed in Komarek *et al.*^1^ (at the Genus level), and in AlgaeBase (Species level), and recorded it in the dataset (Data Citation 1). The same was made to other names by which the strain may be known or to conflicting identifications of co-identical strains. Whenever relevant, we have also added additional taxonomy or nomenclature notes/clarifications to the dataset (e.g., indication of whether it is the Type strain of the holotype).

### Phylogenetic analyses

**Figure 2:**
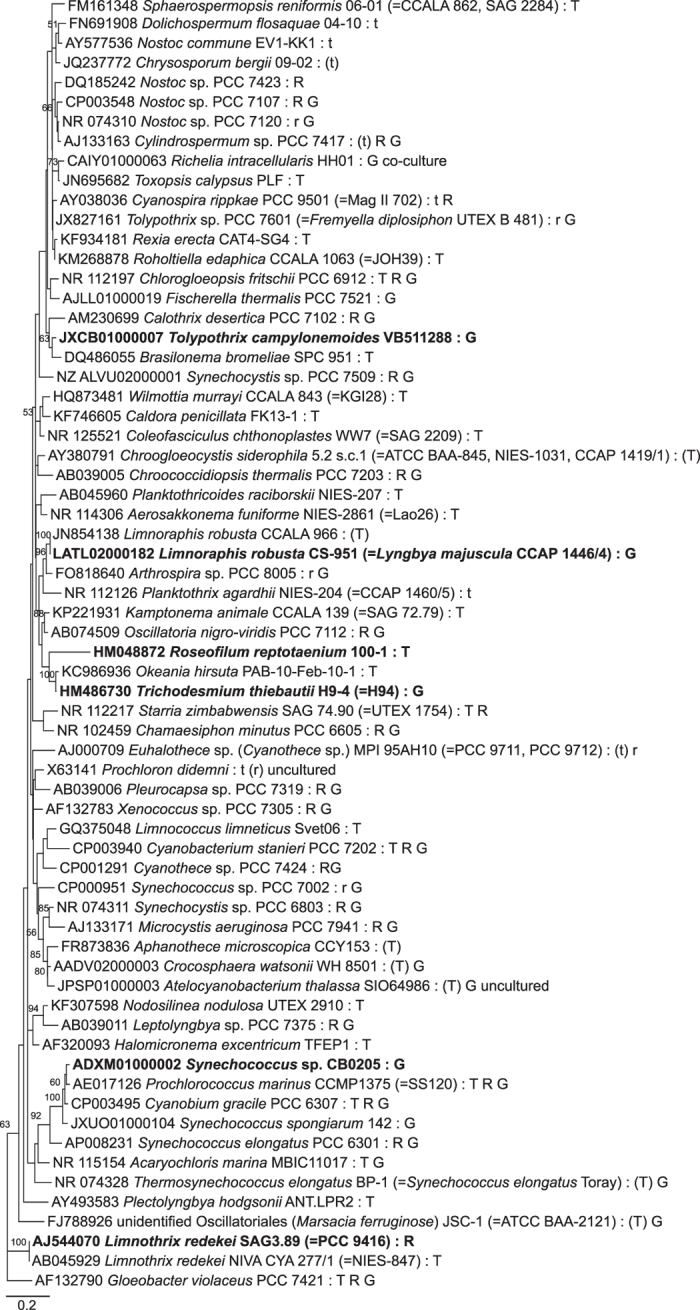
Example of the use of the proposed subset of strains representing the cyanobacterial ‘tree of life’ (see Subset_Condens_Tree in the Data Records section and Phylogenetic analyses in Methods) to evaluate the phylogenetic placement of strains not included in Supplementary Fig. 1 due to having short 16S rRNA gene sequences (in bold). The evolutionary history was inferred by using the Maximum Likelihood method based on the GTR+G+I model. Bootstrap values indicated near internal branches; values below 50% were omitted. Information for each cyanobacterial strain include accession number of the nucleotide sequence, strain ID, eventual taxonomic synonyms or other strain names (in parentheses), and co-identical strains or other strain codes (in parentheses). Letters after colon indicate the categorization of strains as follows (see also Strain_Category in Data Record section): T, Type strain of the Type species; t, not the type strain, but phylogenetically close-related; R, Reference strain in Bergey's Manual of Systematic Bacteriology^9^; r, not the reference strain, but phylogenetically close-related; G, strain with its genome sequenced and publicly available; E, strain studied from exsiccata. A letter in parentheses means that there is a taxonomic-related uncertainty with the taxon name (see taxonomic comments) or the assigned strain’s category couldn’t be yet fully confirmed (e.g., for provisional species names). The outgroup was pruned from the tree for clarity. The scale bar represents nucleotide substitutions per site.

All bioinformatics procedures and analyses were conducted using the MEGA7 software package^17^. Sequences were aligned using the ClustalW algorithm. Strains with small-sized sequences (<1,000 nt) were treated separately, to avoid reducing the number of unambiguously aligned nucleotide positions, and thus preventing distortion of the main phylogeny. Molecular phylogenetic analyses were inferred by using the Maximum Likelihood (ML) method, based on the nucleotide substitution model that best fit the alignment data. By applying the corrected Akaike’s Information Criterion (AICc), the chosen nucleotide substitution model was General Time Reversible (GTR) for both analyses. A discrete Gamma distribution ([+G]) was used to model evolutionary rate differences among sites, while the rate variation model allowed for some sites to be evolutionarily invariable ([+I]). The trees with the highest log likelihood (−27524.3318 and −7982.8912, respectively) are shown for the main (Supplementary Fig. 1) and complementary (Fig. 2) phylogenies. Both trees were rooted with the outgroup *Chloroflexus aurantiacus* J-10-fl (NR_074263).

The phylogenetic analysis for the main tree (Supplementary Fig. 1) involved 333 nucleotide sequences. This figure is available online only. All positions containing gaps and missing data were eliminated. The final alignment dataset consisted of 863 positions. In order to systematize the phylogenetic placement of the cyanobacterial strains, we have grouped the strains into clusters (broader groups; for sequences placed together but lacking bootstrap support) and in clades (for groups of sequences with a ML bootstrap support), as depicted in Supplementary Fig. 1. This primary data was included in the dataset (see also Phylog_This_Work, in the Data Records section). We have also described the phylogenetic placement of the strains according to a selection of important studies^18–23^ (Data Citation 1).

In turn, the analysis performed for the complementary phylogenetic tree (Fig. 2) involved 67 nucleotide sequences. This tree is meant to show the placement of those shorter sequences that were not included in the main tree (six sequences, ranging from 381 to 898 nt; three strains with sequences <315 nt were discarded from the analysis). To do so, we have also included 60 cyanobacterial sequences used in the main tree. The selection of strains involved representatives from all the clades identified in Supplementary Fig. 1 (for larger clades, we have selected a number of divergent strains), intending to cover the cyanobacterial diversity contained in CyanoType v.1. Due to the inclusion of short sequences, less than 5% gaps, missing data, and ambiguous bases were allowed at any position of the alignment. This resulted in a total of 533 positions in the final alignment.

The survey and collection of the different data or metadata (including important bibliographic references for each strain) was finished by the end of 2015, for this version of the database (Data Citation 1).

## Data Records

The dataset, the sequence alignments and the tree files (Data Citation 1) obtained in this work are deposited at FigShare.

The dataset (CyanoType_data_v1.0.csv) is a semi-comma separated values file containing taxonomic- and phylogenetic-related data and other useful information for each cyanobacterial strain considered in this work, including important strain-related references when available (e.g., literature for strain origin, identification/characterization, taxonomy, phylogeny and/or genome sequencing). Rows represent single strains, for which data were integrated. Columns are for useful information and metadata, as follow:

### Entry_number

It is the entry number of the cyanobacterial strain in the dataset.

### Strain_ID

Taxon name and strain code.

### Strain_Other_ID

Other taxon name(s) previously assigned to the strain, synonym(s) of the taxon name, or other putative taxonomic designation(s).

### Strain_Co-Ident

Older code(s) for the strain, misspellings or code(s) from co-identical strains (e.g., same strain deposited in other(s) culture collection(s); not an exhaustive list).

### Strain_Category

Categorization of strains by relevance, as defined in this work, and additional important strain characterization. T, Type strain of the Type species (taxon established by modern polyphasic taxonomy); t, not the type strain but known to have the same phylogenetic placement as the Type species, after taxonomic revision; R, Reference strain in Bergey's Manual of Systematic Bacteriology^9^; r, strain known to be included in the same phylogenetic cluster as the reference strain, as mentioned in the Bergey's Manual^9^; G, strain with its Genome sequenced and publicly available; E, strain studied from Exsiccata (dried herbarium specimens of cyanobacteria). A letter in parentheses means that there is a taxonomic-related uncertainty with the taxon name (see taxonomic comments) or the assigned strain’s category could not be satisfactorily confirmed (e.g., for unpublished, provisional species names).

### Strain_Addition

Additional characterization of the strain concerning its isolation status. ‘Co-culture’ is for strains in culture but associated with other organism (i.e., not free-living isolates).

### Environment

Type of environment from which the strain was obtained.

### Habitat_notes

Additional details on the source/origin or lifestyle of the strain.

### 16S_Acc_Nbr

GenBank accession number for the 16S rRNA gene sequence.

### NCBI_ID

NCBI Assembly or BioProject numbers for available cyanobacterial genomes.

### Tax_Komarek_Ord_Fam

Order and family assignments for the strain identification(s), according to the recent classification scheme proposed by Komárek *et al.*^1^

### Tax_Status_Genus

Status of the genus as depicted from Appendix 1 in Komárek *et al.*^1^, as follows: 1—genera supported by a molecular phylogeny, including a 16S rRNA gene sequence of the type species; 2—genera, from which only one or a few species were studied using molecular methods and for which there is no 16S rRNA gene data for the type species; 3—genera studied using molecular methods and found to be poly/paraphyletic or with no clear relationship with other genera; 4—genera not yet studied using molecular methods; 5—genera not yet validly described; *16*S*-Type*—genera for which there is a 16S rRNA sequence for the type material publicly available (in parentheses, when this availability is not indicated in Komárek *et al.*^1^); problematic genera from the taxonomic point of view.

### Tax_AlgaeBase_Ord_Fam

Order and family assignments for the strain identification(s), according to the online database AlgaeBase (http://www.algaebase.org/).

### Tax_Status_AlgaeBase_&_Tax_Notes

Status of the strain’s taxon name as present in AlgaeBase (http://www.algaebase.org/). When applicable, we indicate whether it is a type strain (i.e., holotype or epitype). It might also include other primary data, such as taxonomic relevant comments or notes.

### Tax_AlgaeBase_Holotype

Type species of the genus (holotype) and authority as indicated in AlgaeBase (http://www.algaebase.org/). It may include some additional taxonomic relevant comments or notes.

### Tax_Bergey's

Classification according to the Bergey's Manual scheme^9^, in condensed form. The first roman numerals refer to subsections, while the second refer to form-genus within that subsection.

### Phylog_This_Work

Position of the strain within the phylogenetic tree illustrated in Supplementary Fig. 1 (capital letters and numbers refer to clusters and clades, respectively).

### Subset_Condens_Tree

Subset of 60 strains for a proposal of a condensed phylogenetic tree covering the cyanobacterial diversity included in CyanoType (see also Fig. 2 and the Subsection ‘Phylogenetic analyses’ in Methods). The goal of this suggested subset is to aid users in preliminary phylogenetic analyses, namely to discern the placement of their sequences in relation to relevant strains.

### Phylog_RDP_Classifier

Classification according to the automatic RDP Naive Bayesian rRNA Classifier^16^.

### Phylog_Shih

Phylogenetic placement of the strain (clade or sub-clade) as established in Shih *et al.*^14^

### Phylog_Howard-Azzeh

Phylogenetic placement of the strain (clade or sub-clade) as established in Howard-Azzeh *et al.*^19^

### Phylog_Schirrmeister

Phylogenetic placement of the strain (clade or sub-clade) as established in Schirrmeister *et al.*^19^

### Phylog_Picocyano

Ecotypes as established or present in Ahlgren & Rocap^20^, Ahlgren *et al.*^21^, Kettler *et al.*^22^ or Scanlan *et al.*^23^. For *Prochlorococcus* and *Synechococcus* spp. strains only.

### Metadata_Shih

Information for additional metadata present in Shih *et al.*^14^.

### References

Important literature related with the strain (e.g., with information on isolation/source origin, identification/taxonomy, phylogeny, genome sequencing, etc.).

## Technical Validation

The dataset was extensively checked for double entries, errors or inconsistencies (all fields), while data or metadata concerning each entry (i.e., strain) was further revised, very particularly decisions about category attribution (see Fig. 1). Whenever available, bibliographic references are provided for each entry, enabling any user to get access to the original data. Researchers making use of the dataset (Data Citation 1) or the database are encouraged to assess the validity and accuracy of the data and send us feedback through the website database, at http://lege.ciimar.up.pt/cyanotype. The information will be updated after curation by our team.

In the future, it is intended that the information for any given entry (i.e., strain) in the database may be curated on a voluntary basis. To this end, administrative and managerial procedures for quality control of data will be implemented. For instance, users will need to request permission to become a contributor and will have a user account. Any observation made by a contributor will be flagged and simultaneously an automatic message will be sent to the administrator. The ‘pending’ flag will be removed only after administrator approval. The observation made by the contributor for a particular strain will be then recorded and become accessible to other users, as updated information for that strain.

## Additional Information

**How to cite this article:** Ramos, V. *et al.* A curated database of cyanobacterial strains relevant for modern taxonomy and phylogenetic studies. *Sci. Data* 4:170054 doi: 10.1038/sdata.2017.54 (2017).

**Publisher’s note:** Springer Nature remains neutral with regard to jurisdictional claims in published maps and institutional affiliations.

## Supplementary Material



Supplementary Figure 1

## Figures and Tables

**Table 1:** Number of cyanobacterial strains included in version 1 of the CyanoType dataset and present in the phylogenetic trees obtained in this study, by category: T, Type strain of the Type species; t, not the type strain, but phylogenetically close-related; R, Reference strain in Bergey's Manual of Systematic Bacteriology^9^; r, not the reference strain, but phylogenetically close-related; G, strain with its genome sequenced and publicly available; E, strain studied from exsiccata.

Strain category*	**# of strains**	**# of strains included in the provided phylogenetic trees**
T or t, only	73	63
T or t and R or r	5	4
T or t and G	10	10
T or t and R or r and G	9	9
R or r and G	60	60
R or r, only	41	30
G, only	172	155
E	1	1
TOTAL	371	332

*see also categories descriptions in the Data Records section.
