# Supplementary figures and images for: A curated database of cyanobacterial strains relevant for modern taxonomy and phylogenetic studies
Source: Sci Data. 2017 Apr 25;4:170054. doi: 10.1038/sdata.2017.54 (PMC5404626; doi:10.1038/sdata.2017.54)

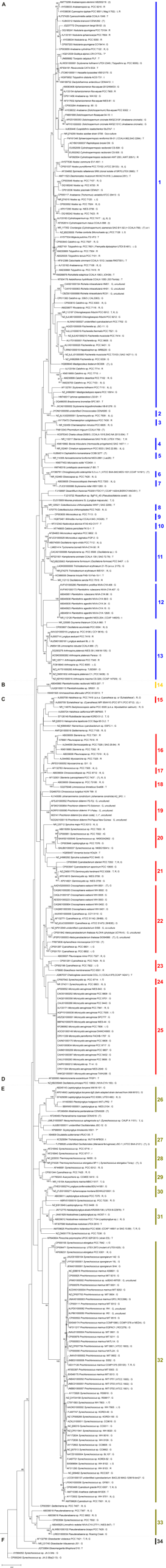

Supplement: Supplementary Figure 1 [file sdata201754-s2.pdf]
